# Multidimensional Sleep Health Methods: Findings From US and Netherlands Cohorts of Older Adults

**DOI:** 10.1093/geroni/igaf122.771

**Published:** 2025-12-31

**Authors:** Meredith Wallace

**Affiliations:** University of Pittsburgh, Pittsburgh, Pennsylvania, United States

## Abstract

Sleep health is a multidimensional construct, with dimensions including Regularity, Satisfaction, Alertness/Sleepiness, Timing, Efficiency, and Duration. Global estimates from large cohort studies suggest that approximately 30-40% of adults report ≥2 suboptimal sleep health dimensions. Moreover, poor sleep health dimensions are established risk factors for disease and disability, regardless of the presence or absence of a sleep disorder. Although current literature indicates that different sleep health dimensions are associated with different health outcomes, several methodological challenges have created hurdles to understanding which combinations of sleep dimensions are most relevant for each health outcome. In this presentation, Dr. Wallace will outline and demonstrate approaches for quantifying multidimensional sleep health and relating it to health outcomes. These methods range from the simple and clinically practical (e.g., using simple self-reported sleep health questionnaires) to the statistically complex (e.g., machine learning models that empirically derive weights to optimally combine sleep health features; or empirically deriving common within-person combinations of sleep health dimensions). Dr. Wallace will compare, contrast and demonstrate these methods using an international multi-cohort sample of older adults from the United States and the Netherlands, including data from the Osteoporotic Fractures in Men Study, the Study of Osteoporotic Fractures, the Multi-Ethnic Study of Atherosclerosis, the Rush Memory and Aging Project, Minority Aging Research Study, and the Rotterdam Study. With these data and methods, she will discuss the role of sleep health in relation to longitudinal trajectories of depression and cognitive outcomes.

